# Corrigendum: Abnormalities of thymic stroma may contribute to immune dysregulation in murine models of leaky severe combined immunodeficiency

**DOI:** 10.3389/fimmu.2024.1467807

**Published:** 2024-09-05

**Authors:** Francesca Rucci, Pietro Luigi Poliani, Stefano Caraffi, Tiziana Paganini, Elena Fontana, Silvia Giliani, Frederick W. Alt, Luigi Daniele Notarangelo

**Affiliations:** ^1^ Division of Immunology and The Manton Center for Orphan Disease Research, Children’s Hospital Boston, Boston, MA, United States; ^2^ Department of Pathology, University of Brescia, Brescia, Italy; ^3^ “Angelo Nocivelli” Institute for Molecular Medicine and Department of Pediatrics, University of Brescia, Brescia, Italy; ^4^ Howard Hughes Medical Institute, Children’s Hospital, Immune Disease Institute and Harvard Medical School, Boston, MA, United States

**Keywords:** severe combined immunodeficiency, recombination-activating gene 1, DNA ligase 4, thymic epithelial cells, thymus, dendritic cells, Aire, regulatory T cells

In the published article, there was an error in **Figure 5** as published.

“The insets within the right upper and middle panels showing a higher magnification of the AIRE immunostaining have been corrected. In the original panels, there was a mistake in placing the original insets from a higher magnification image.”

The corrected **Figure 5** and its caption appear below.

**Figure 5:**
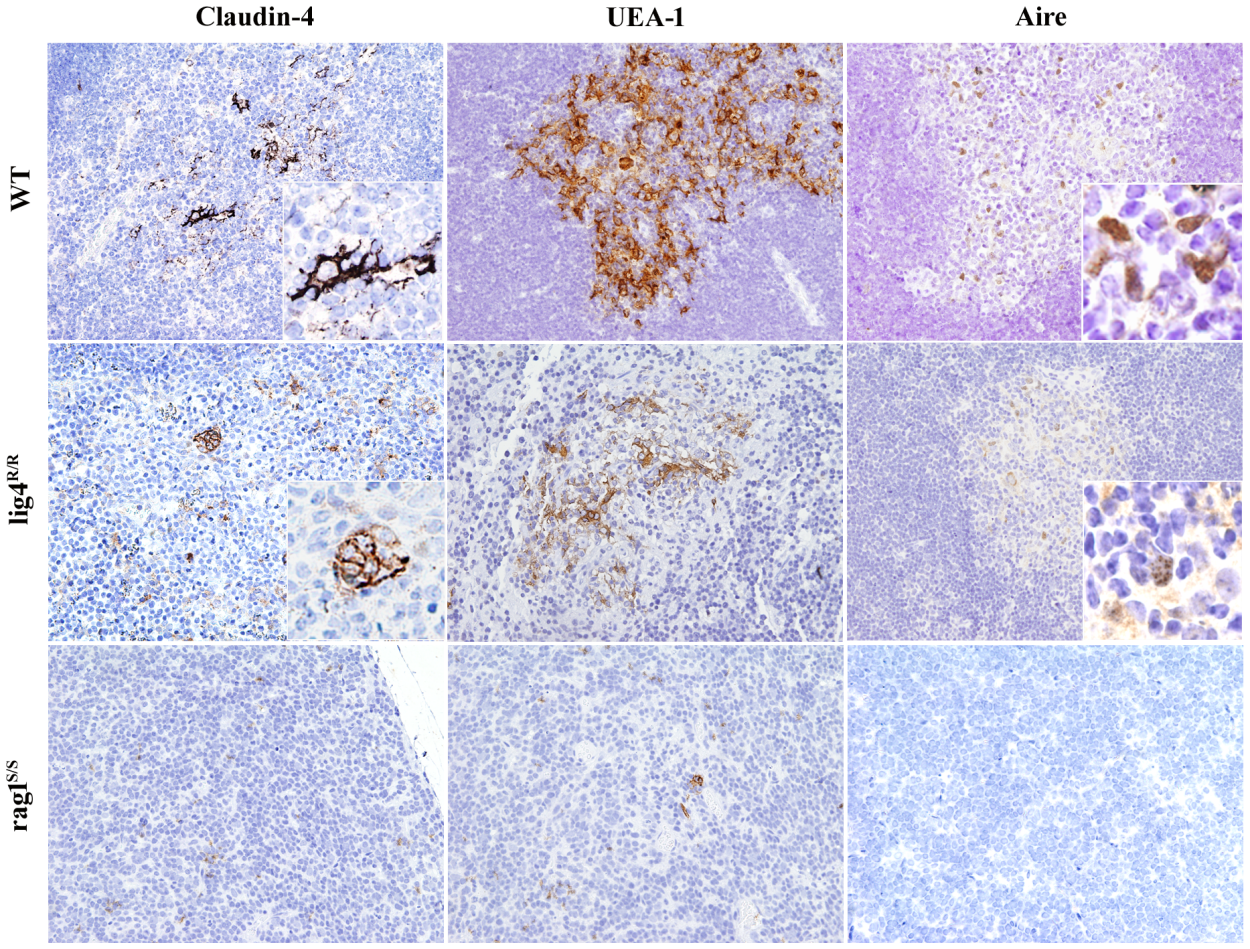
Maturation of medullary thymic epithelial cells (mTECs) in wild-type (WT), *Lig4* ^R/R^, and *Rag1* ^S/S^ mice. Mature mTECs from WT mice express claudin-4 (Cld4), Ulex europaeus agglutinin 1 (UEA-1) and Aire (upper panels). Insets highlight fully mature mTECs showing immunoreactivity (IR) for Cld4 and the characteristic granular dot-like Aire positivity in the nuclei. Thymuses from *Lig4* ^R/R^ mice show residual presence of mTECs that reach full maturation with positivity for UEA-1, Cld4, and Aire expression (middle panels). Loss of corticomedullary demarcation (CMD) with impaired maturation of mTECs was observed in the thymuses from the *Rag1* ^S/S^ mice in which only rare UEA-1 IR cells but no mature Cld4^+^ and Aire^+^ cells were found (lower panels). IR staining: brown. All panels are from 20× original magnification; insets are from 40× original magnification. One representative example of five mice analyzed per each strain.

The authors apologize for this error and state that this does not change the scientific conclusions of the article in any way. The original article has been updated.

